# Systematic alteration of ATAC-seq for profiling open chromatin in cryopreserved nuclei preparations from livestock tissues

**DOI:** 10.1038/s41598-020-61678-9

**Published:** 2020-03-23

**Authors:** M. M. Halstead, C. Kern, P. Saelao, G. Chanthavixay, Y. Wang, M. E. Delany, H. Zhou, P. J. Ross

**Affiliations:** Department of Animal Science, University of California, Davis, Davis, CA USA

**Keywords:** Chromatin analysis, Data acquisition

## Abstract

The use of Assay for Transposase-Accessible Chromatin (ATAC-seq) to profile chromatin accessibility has surged over the past years, but its applicability to tissues has been very limited. With the intent of preserving nuclear architecture during long-term storage, cryopreserved nuclei preparations from chicken lung were used to optimize ATAC-seq. Sequencing data were compared with existing DNase-seq, ChIP-seq, and RNA-seq data to evaluate library quality, ultimately resulting in a modified ATAC-seq method capable of generating high quality chromatin accessibility data from cryopreserved nuclei preparations. Using this method, nucleosome-free regions (NFR) identified in chicken lung overlapped half of DNase-I hypersensitive sites, coincided with active histone modifications, and specifically marked actively expressed genes. Notably, sequencing only the subnucleosomal fraction dramatically improved signal, while separation of subnucleosomal reads post-sequencing did not improve signal or peak calling. The broader applicability of this modified ATAC-seq technique was tested using cryopreserved nuclei preparations from pig tissues, resulting in NFR that were highly consistent among biological replicates. Furthermore, tissue-specific NFR were enriched for binding motifs of transcription factors related to tissue-specific functions, and marked genes functionally enriched for tissue-specific processes. Overall, these results provide insights into the optimization of ATAC-seq and a platform for profiling open chromatin in animal tissues.

## Introduction

Complex multicellular animals are made up of an immense variety of cell types that vary physiologically and functionally, despite sharing the same genomic blueprint. This assortment of cell types is largely explained by differences in gene expression, meaning that cell types demonstrate unique expression profiles, or transcriptomes, that result from complex transcriptional regulation by functional elements in the genome. Previous efforts to annotate these regulatory elements, such as the Encyclopedia of DNA Elements (ENCODE) projects, have used a range of genomic assays to characterize the epigenomes of humans and classical model organisms^1–5^ to identify regulatory elements and their activities in a variety of tissues and cell types. The DNA element atlases generated by these projects have subsequently proved invaluable to research, improving our basic understanding of genome organization and gene regulation, facilitating detection of causative variants, and allowing interspecies comparison of regulatory programs. The latter has revealed that regulatory mechanisms and expression patterns have substantially diverged between species^2^, highlighting the need to annotate functional regulatory regions in additional organisms— particularly those of economic value, such as livestock.

The Functional Annotation of Animal Genomes (FAANG) initiative intends to address this gap in genome annotation in animal species. One of the core goals of this initiative is to standardize the genomic assays that are used to identify regulatory elements. Since open chromatin facilitates the DNA-protein interactions that underlie DNA element functionality, methods for profiling chromatin accessibility are essential for identifying functional regions. In large part, ENCODE used DNase-I hypersensitive sites sequencing (DNase-seq) to detect open chromatin^6^. However, the recently developed Assay for Transposase-Accessible Chromatin (ATAC-seq) constitutes an attractive alternative to DNase-seq, largely due to its simplicity and low input requirements^7–9^. Thus far, ATAC-seq has most commonly been used to profile open chromatin in cultured cells and has not yet been broadly applied to tissues or frozen samples. In the context of the FAANG initiative, this necessitates the development of a robust modified ATAC-seq protocol that is compatible with stored tissue samples.

Recent changes to the ATAC-seq protocol suggest that snap-frozen tissues may be used to profile chromatin accessibility^10^, but it has also been shown that flash-frozen cultured cells were unsuitable for ATAC-seq, suggesting that the flash-freezing process may impair nuclear architecture^11^. Alternatively, it was shown that cryopreservation by slow-cooling resulted in an open chromatin profile similar to that obtained from fresh cells^11^.

To this end, a nuclei isolation and cryopreservation protocol initially developed for use with DNase-I treatment was employed to generate cryopreserved nuclei preparations from freshly collected tissue. To take advantage of pre-existing genomic data on chicken lung, cryopreserved nuclei preparations were generated from fresh lung tissue harvested from inbred adult roosters. Using these samples, the original ATAC-seq protocol was systematically modified with the intent of producing high-quality sequencing data that demonstrated strong signal and correlation with supplementary genomic data sets. Regions of open chromatin were expected to correlate with local gene expression, active histone marks, and DNase I hypersensitive sites (DHS); therefore, ATAC-seq data were compared with existing DNase-seq (Stam Lab, University of Washington), Chromatin Immunoprecipitation sequencing (ChIP-seq), and RNA-seq data generated for adult male chicken lung as part of the FAANG pilot project at the University of California, Davis (UCD).

**Figure 1:**
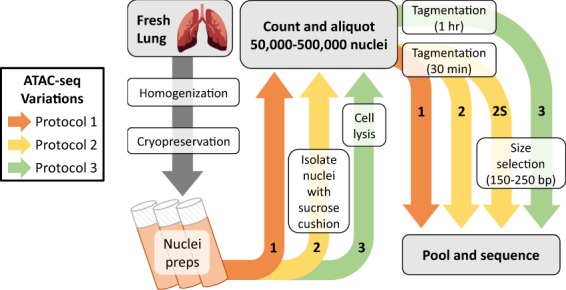
ATAC-seq optimization process for compatibility with cryopreserved nuclei preparations. Variations to the ATAC-seq protocol are labeled chronologically, with the first iteration (1) shown in orange, the second iteration (2) in yellow, the second iteration with a size-selection step (2S) also in yellow, and the third iteration (3) in green.

Briefly, the original ATAC-seq protocol^8^ involves (1) cell lysis, (2) tagmentation with hyperactive Tn5 transposase, which simultaneously cuts DNA at accessible regions and inserts sequencing adapters, and (3) PCR amplification, followed by sequencing. Modifications to each step were explored methodically to optimize sequencing data quality (Fig. 1), revealing that with specific modifications, the ATAC-seq can be made compatible with cryopreserved nuclei preparations. These results broaden the applicability of ATAC-seq to samples that were otherwise outside the scope of the original method, including those that are indispensable to the FAANG community.

## Results

### First iteration of ATAC-seq from cryopreserved nuclei preparations yields poor signal

**Figure 2:**
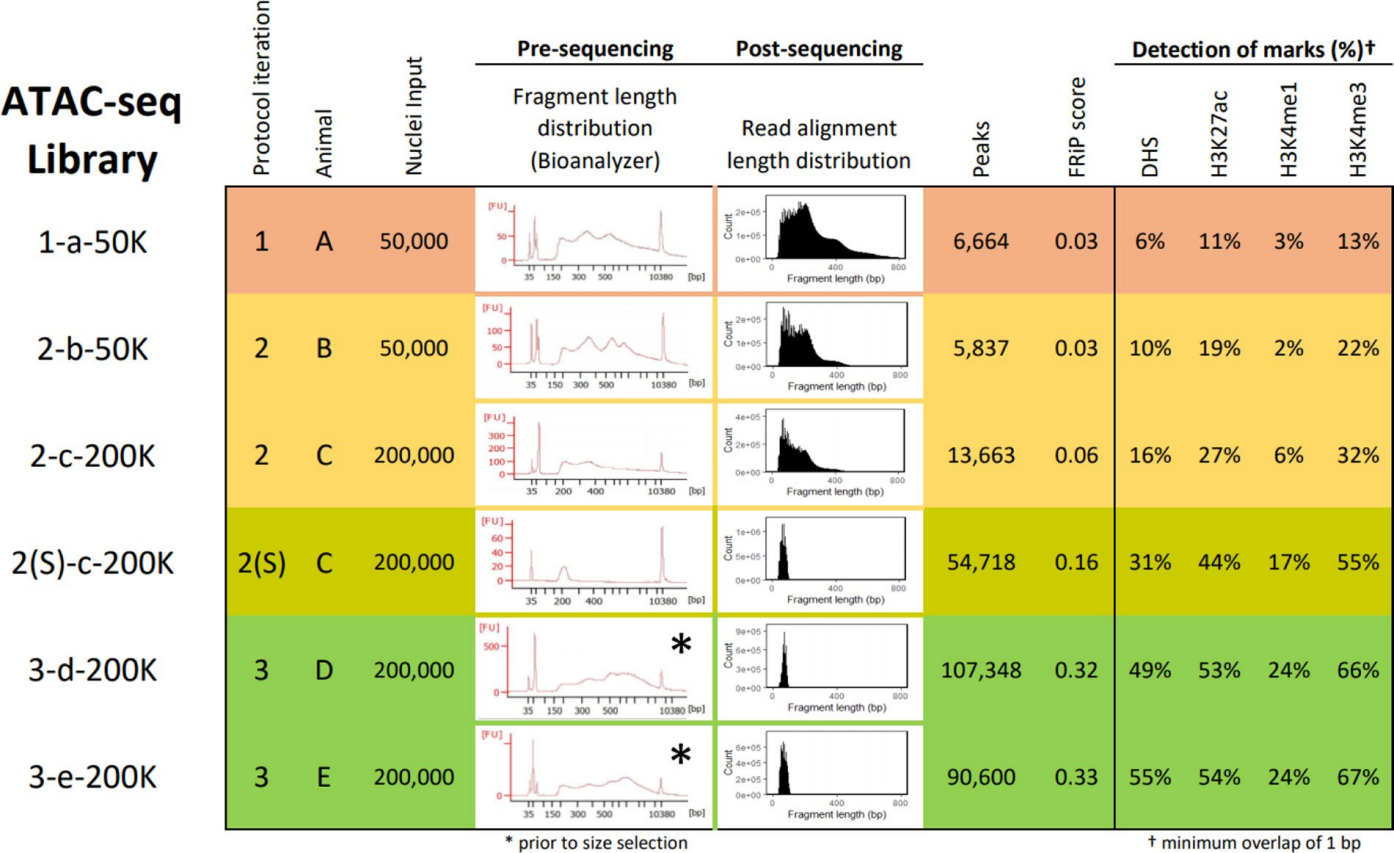
Summary of representative libraries for each ATAC-seq protocol iteration, produced from cryopreserved chicken lung nuclei. Row colors indicate different iterations of the ATAC-seq protocol. Library names indicate “Protocol iteration–Animal–Nuclei input(–Replicate)”. Progressive changes to the protocol improved signal (Fraction of Reads in Peaks (FRiP) score) and improved overlap with DNase-I Hypersensitive Sites (DHS) and active histone modifications H3K27ac, H3K4me1, and H3K4me3 (minimum 1 bp overlap).

**Figure 3:**
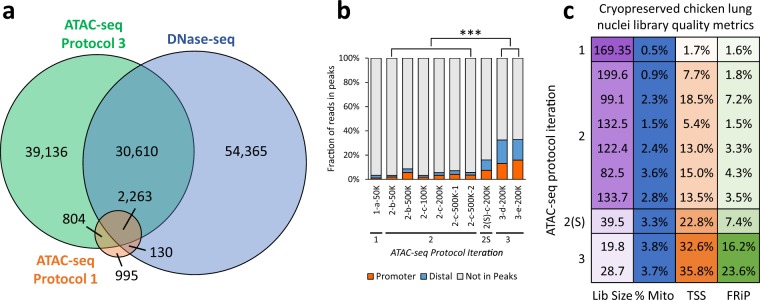
Comparison of different iterations of the ATAC-seq protocol with DNase-seq. (**a**) Overlap of chicken lung peaks called from 30 million reads derived from DNase-seq and ATAC-seq protocols 1 and 3 (libraries 1-a-50K and 3-e-200K) (minimum 1 bp overlap). (**b**) Fraction of reads in peaks (FRiP) that overlap (minimum 1 bp) promoters (±2 kb from TSS) and distal elements (>2 kb from TSS) from libraries generated using different iterations of ATAC-seq; ***P < 0.001 by two-tailed unpaired Student’s t-test comparing the fraction of reads in peaks to reads outside of peaks. All values were determined from 40 million random aligned de-duplicated reads. (**c**) ATAC-seq quality metrics: estimated library size (“Lib size”; purple), percentage of reads that mapped to mitochondrial DNA (“% Mito”; blue), enrichment of signal at promoters (2 kb upstream from TSS; orange; Ensembl Galgal5.0.94 annotation; minimum 1 bp overlap of ATAC-seq peak with promoter), and FRiP (green). Desirable values are shaded darker, with the scale starting at 0 (white) and ending at the maximum value. For the percent mitochondrial reads, anything below 5% is shaded blue. All values were determined from 5 million random aligned reads.

Fresh lung tissue collected from adult inbred roosters was homogenized, filtered to remove debris, supplemented with cryoprotectant and slow-frozen. This method is intended to isolate nuclei from tissues for cryopreservation, before treatment with DNase-I^12^. Following a similar methodology for use with DNase-seq, cryopreserved nuclei preparations were thawed on ice, pelleted, resuspended in sucrose buffer, filtered, pelleted again, washed, and resuspended in PBS for counting. At this point, the original ATAC-seq protocol^8^ was followed without modification. Briefly, 50,000 nuclei were counted, pelleted, and incubated for 30 minutes with hyperactive Tn5 transposase. Transposed DNA was then amplified by PCR (with 12 total cycles determined as optimal by qPCR) and inspected for fragment length distribution on a Bioanalyzer High Sensitivity DNA Chip (Agilent Genomics). This revealed the expected nucleosomal laddering pattern, with subnucleosomal, mononucleosomal, and dinucleosomal fragments enriched at 200, 350, and 550 bp, respectively (Fig. 2; Library *1-a-50K*). Taking this as an indication that the genomic DNA had been successfully tagmented, the library was submitted for sequencing, resulting in over 137 million uniquely mapped monoclonal non-mitochondrial reads (Table 1). However, at a depth of 40 million reads, only 6,664 regions showed significant enrichment, and only 6% of DHS were detected (Figs. 2 and 3a).

**Table 1:** Summary of raw sequence data, read alignment, and read filtering for all ATAC-seq libraries. Names of chicken lung libraries indicate “Protocol iteration–Animal–Nuclei input(–Replicate)”.

Species	Tissue	Library	Raw reads	Mapped	Mitochondrial	Duplicates	Available to call peaks	
				(% raw)	(% mapped)	(% mapped)	(% mapped)	
Chicken	Lung	1-a-50K	196,278,926	92.1	0.5	20.4	137,101,850	(75.8%)
		2-b-50K	96,657,284	95.8	0.9	9.5	79,259,427	(85.6%)
		2-b-500K	74,137,422	93.9	2.3	12.0	57,757,465	(83.0%)
		2-c-100K	102,137,944	95.7	1.4	14.1	79,044,086	(80.9%)
		2-c-200K	123,755,468	94.8	2.4	14.9	93,751,696	(79.9%)
		2-c-500K-1	114,037,600	94.4	3.6	17.7	82,925,421	(77.1%)
		2-c-500K-2	93,624,326	95.0	2.8	9.9	75,192,492	(84.6%)
		2S-c-200K	183,267,356	92.7	3.3	43.5	89,022,914	(52.4%)
		3-d-200K	111,908,958	91.9	3.8	51.5	45,522,369	(44.3%)
		3-e-200K	110,665,462	91.3	3.7	36.3	59,472,019	(58.9%)
Pig	Lung	A	107,974,450	82.1	6.3	20.7	57,559,985	(64.9%)
		B	110,602,142	84.3	6.9	18.5	58,731,355	(63.0%)
	Muscle	A	167,952,108	89.4	5.6	26.5	88,959,417	(59.3%)
		B	140,846,406	89.3	4.1	29.5	72,530,199	(57.7%)
	Spleen	A	91,491,880	85.6	3.2	16.7	50,900,468	(65.0%)
		B	93,647,288	86.4	2.2	15.8	53,024,747	(65.6%)

Data were generally noisy, with reads aligning broadly across the genome rather than accumulating at DHS. The Fraction of Reads in Peaks (FRiP) was used to measure the signal-to-noise ratio, since reads falling within regions of enrichment are informative of open chromatin, whereas those falling outside those regions are background noise. In this case, the FRiP score was 0.06, well beneath the minimum 0.2 standard set by ENCODE for ATAC-seq libraries (Fig. 3b). This uniform distribution clearly contradicted the nucleosomal laddering pattern that was observed prior to sequencing, which suggested that tagmentation had resulted in enrichment for fragment lengths that should have specifically corresponded to either open chromatin, or DNA wrapped around one or more nucleosomes. It was speculated that the poor library quality of *1-a-50K* could have resulted from either (1) degraded cells in the nuclei preparations, whose free-floating genomic DNA was contributing the bulk of ATAC-seq reads, or (2) under-tagmentation, indicating that either the incubation period with the transposase was too short, or the number of cells used in the reaction too high.

### Varying nuclei input does not substantially improve ATAC-seq library quality

To exclude cell debris from the tagmentation reaction, intact nuclei were isolated from cryopreserved nuclei preparations using a sucrose cushion and ultracentrifugation. Preparations were thawed on ice, carefully layered on top of a sucrose gradient and pelleted. Nuclei were then counted and ATAC-seq libraries were prepared as previously. All libraries were PCR amplified for a total of 10 cycles – two less than the optimal number of PCR cycles for *1-a-50K* – to minimize PCR bias. The number of nuclei per transposition reaction was varied from 50,000 to 500,000 to determine if over- or under-transposition was affecting the signal-to-noise ratio after sequencing.

**Figure 4:**
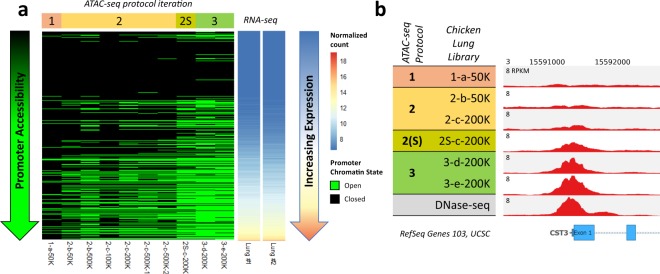
Relationship between gene expression and promoter chromatin accessibility in chicken lung for different ATAC-seq protocol iterations. (**a**) All genes from the Galgal5 Ensembl 94 annotation were sorted by expression (normalized VST counts) and their promoters (±2 kb from TSS) were classified as either open (green) or closed (black), based on overlap with ATAC-seq peaks from each chicken lung library (minimum overlap of 1 bp). RNA-seq data were procured from different individuals than the ATAC-seq data. (**b**) Normalized read depth of ATAC-seq and DNase-seq libraries at the TSS of the highly expressed gene cystatin C (CST3).

The rate of duplication among mapped reads remained below 20% (Table 1), indicating that PCR bias remained below the level that was introduced when PCR cycling was initially optimized. After randomly subsampling all libraries to a depth of 40 million reads for peak calling, 15% of DHS were detected on average by ATAC-seq peaks (Fig. 2 and Supplementary Fig. 1; Protocol Iteration 2), with an average FRiP score of 0.06 (Fig. 3b). Detection of open chromatin at transcription start sites (TSS) was also improved. Open chromatin identified by *1-a-50k* only marked 1.7% of promoters, whereas open chromatin identified by libraries from the second ATAC-seq iteration marked 12% of promoters, on average (Fig. 3c), with preference for actively expressed genes (Fig. 4). While these libraries demonstrated slight improvements over *1-a-50K*, they were still not of sufficient quality. Detection of DHS remained below the 46% detected by the original ATAC-seq protocol in a cell line^8^, and signal was still far beneath the 0.2 minimum FRiP score required by ENCODE.

As observed in the first ATAC-seq iteration (*1-a-50K*), the signal-to-noise ratio remained too low to reliably distinguish peaks in regions with DHS, despite clear nucleosomal laddering patterns prior to sequencing. In addition, isolation of subnucleosomal length alignments (observed template length <150 bp) for peak calling did not improve signal or specificity of reads to DHS. Linear regression analysis of all ATAC-seq libraries from the second protocol iteration showed that higher FRiP scores were correlated with improved detection of DHS (R^2^ = 0.83, p = 0.01), indicating that a high signal-to-noise ratio is critical to library quality. Increasing the number of nuclei used in the transposition reaction did not significantly impact number of peaks called (R^2^ = 0.55, p = 0.09), but appeared to somewhat improve the FRiP score (R^2^ = 0.74, p = 0.03) and DHS detection (R^2^ = 0.82, p = 0.01). Despite issues with low signal, 7,546 peaks (61%) were shared between technical replicates (libraries *2-c-500K-1* and *2-c-500K-2*), and 6,841 peaks (55%) were shared between biological replicates (libraries *2-b-500K* and *2-c-500K-2)*.

Overall, it was concluded that (1) nucleosomal laddering was not a reliable indicator of library quality, (2) isolating nuclei by sucrose gradient did not solve the issue of low signal, (3) isolating subnucleosomal length reads post-sequencing also did not solve the issue of low signal, and (4) increasing nuclear input could somewhat improve signal quality and DHS detection.

### Sequencing only the subnucleosomal fraction substantially improves ATAC-seq signal

Pursuing the possibility that there was some low-level enrichment of subnucleosomal reads at TSS, a library with sufficient material for re-sequencing (*2-c-200K*) was size-selected for only subnucleosomal length fragments (150–250 bp) using the PippinHT system, and then re-sequenced on the NextSeq platform to generate 40 bp paired-end reads. This substantially improved the FRiP score from 0.06 to 0.16 (Fig. 3b), quadrupled the number of peaks called, and nearly doubled the detected DHS from 16% to 31% (Fig. 2; library *2S-c-200K*). Clearly, size-selection of libraries for subnucleosomal length fragments substantially improved library quality.

Postulating that library quality could be further improved, alternative methods for isolating nuclei prior to transposition were explored. Previously, a sucrose gradient separation had been employed to isolate intact nuclei for ATAC-seq. In many cases, a large amount of material did not pellet consistently and was discarded. Reasoning that this material might contain intact cells that had not been lysed during the homogenization step prior to cryopreservation, all material from thawed nuclei preparations were pelleted, washed, and incubated with cell lysis buffer. Considering the correlation between increased nuclei input and signal, 200,000–500,000 nuclei were incubated with transposase. Transposition time was also varied from 30 to 90 minutes, and transposed DNA was amplified for 10 PCR cycles as previously. The fragment length distributions of these libraries shifted considerably in response to varied incubation time; the longer the incubation, the higher proportion of subnucleosomal length fragments (Supplementary Fig. 2). For 200,000 nuclei, 30 minutes of exposure to transposase resulted in a preponderance of high molecular weight fragments, possibly indicating under-transposition. At 90 minutes, laddering after the mononucleosomal peak was lost, suggesting over-transposition. Consequently, nuclei were transposed for 60 minutes total, such that the subnucleosomal fraction and nucleosomal laddering were both prominent. Libraries were size-selected for subnucleosomal length fragments, and submitted for sequencing on the NextSeq 500 platform to generate 40 bp paired-end reads. As expected, since smaller fragments are more efficiently amplified by PCR, size selection for the subnucleosomal fraction increased the rate of PCR duplication in (Table 1), indicating that total PCR cycles should be carefully considered when size-selecting libraries.

After subsampling to a depth of 40 million reads (Fig. 2; Libraries *3-d-200K* and *3-e-200K*), libraries from the third ATAC-seq protocol iteration captured more DHS than previously and demonstrated FRiP scores above 0.3 (Fig. 3a,b). Peak calls were consistent between biological replicates, with 61,503 peaks (67.9%) from *3-e-200K* directly overlapped by *3-d-200K* peaks. These nucleosome-free regions (NFR) also consistently overlapped activating histone modifications (Fig. 2; ChIP-seq data generated as part of the UC Davis FAANG pilot project): H3K27ac (typically a marker of active enhancers; average 54% ChIP-seq peaks detected), H3K4me1 (a general mark of enhancers; average 24% detected), and H3K4me3 (a marker of active TSS; average 67% detected). Additionally, these NFR more consistently marked the promoters of expressed genes compared to data from previous ATAC-seq protocol iterations (Fig. 4).

Comparing the size-selected libraries *2S-c-200K*, *3-d-200K*, and *3-e-200K*, it is evident that the additional lysis step and doubled transposition time improved library quality. At a depth of 40 million uniquely mapped monoclonal non-mitochondrial reads, *2S-C-200K* only detected about a third of DHS, with a FRiP score less than 0.2. In contrast, *3-d-200K* and *3-e-200K* each detected about half of DHS, with FRiP scores over 0.3. Specificity of ATAC-seq peaks to promoters of actively expressed genes was also improved. The promoters of some actively expressed genes were not marked by NFR according to *2S-c-200K*, but this was resolved in *3-d-200K* and *3-e-200K*, wherein most actively expressed genes contained open chromatin in their promoters (Fig. 4a).

To gauge the target sequencing depth for future experiments, all available uniquely-mapped, monoclonal, non-mitochondrial reads were combined from *3-d-200K* and *3-e-200K*, and then randomly subsampled from 10 to 90 million reads. These subsampled read sets were then subjected to peak calling and assessed for DHS detection (Supplementary Fig. 3), revealing a plateau in additional DHS detected after about 50 million mapped reads, in concordance with the recommendations by Buenrostro *et al*. (2013).

### Chicken lung ATAC-seq data marks both promoters of active genes and intergenic regions

**Figure 5:**
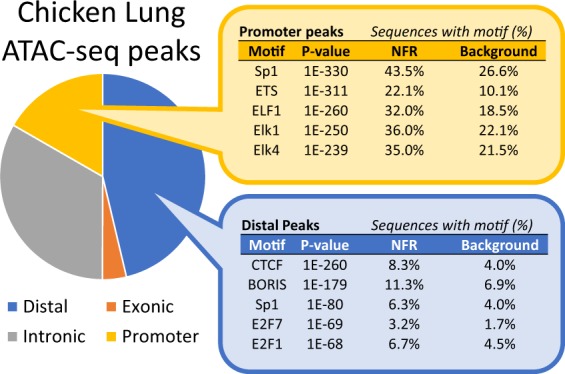
Characterization of the 61,503 chicken lung ATAC-seq peaks that were shared between libraries from the third ATAC-seq protocol iteration. Genomic location of peaks was determined by minimum 50% overlap with promoters (2 kb upstream of TSS), exons, or introns. Peaks that did not overlap any features were classified as distal. The top 5 enriched binding motifs are reported for peaks that were distal or which localized to promoters.

One concern when sequencing only the subnucleosomal fraction was whether this would lead to preferential detection of TSS. Buenrostro *et al*. (2015) showed that TSS were enriched for nucleosome-free reads as compared to distal elements, implying that size selection for the subnucleosomal fraction could lead to an over-representation of TSS in ATAC-seq peaks. In contrast to this expectation, only a third of NFR found in both *3-d-200K* and *3-e-200K* corresponded to promoters (2 kb upstream of TSS), and nearly half fell in intergenic regions (Fig. 5). The NFR that overlapped promoters were most significantly enriched for the binding motif of Sp1, a transcription factor involved in many cellular processes, and that of ETS factors, which are also implicated in a wide variety of functions via transcriptional regulation. Distal NFR were most significantly enriched for the CTCF binding motif, suggesting that many of these peaks are likely involved in the 3-D organization of the genome, since CTCF has been implicated in long-range chromatin interactions, such as those between enhancers and their targets^13^.

### Modified ATAC-seq generates high quality chromatin accessibility data for pig lung, muscle and spleen

**Figure 6:**
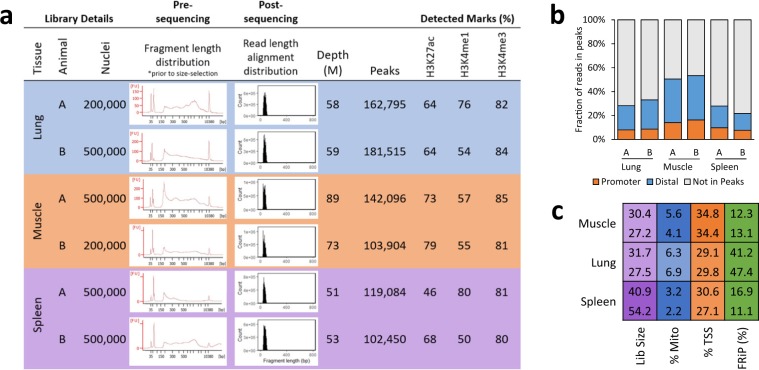
ATAC-seq libraries generated for three adult pig tissues. (**a**) Summary of library details, read depth used for calling peaks, and overlap (minimum 1 bp) with active histone modifications. (**b**) Fraction of reads in peaks (FRiP) that overlapped (minimum 1 bp) promoters (2 kb upstream of TSS) and distal elements (>2 kb from TSS). (**c**) ATAC-seq quality metrics: estimated library size (“Lib size”; purple), percentage of reads that mapped to mitochondrial DNA (“% Mito”; blue), enrichment of signal at promoters (±2 kb from TSS; orange; Ensembl Sscrofa10.2.89 annotation; minimum 1 bp overlap of ATAC-seq peak with promoter), and FRiP (green). Desirable values are shaded darker, with the scale starting at 0 (white) and ending at the maximum value. For the percent mitochondrial reads, anything below 5% is shaded blue. For FRiP, all values above 10% are shaded green. All values were determined from 5 million random aligned reads.

**Figure 7:**
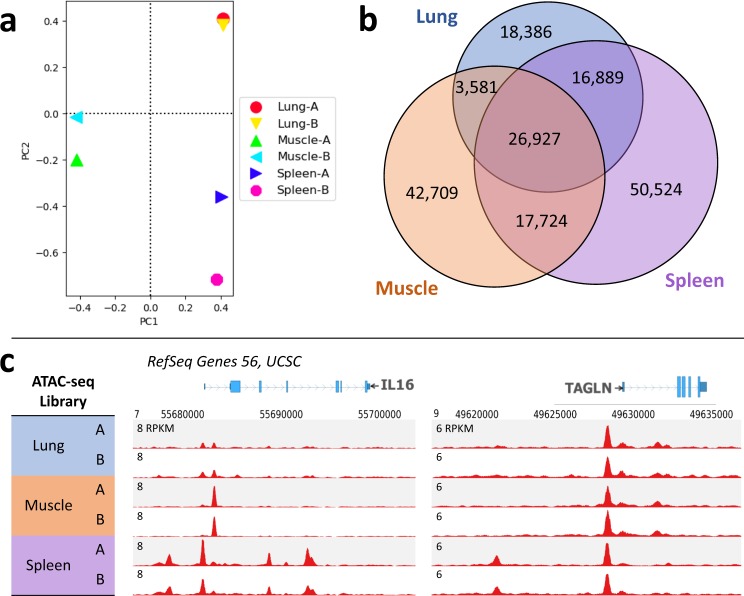
Comparison of adult pig lung, muscle, and spleen chromatin accessibility profiles. (**a**) Principal components analysis of read alignments for each library shows that biological replicates of each tissue cluster together. (**b**) Overlap of ATAC-seq peaks that were detected in both biological replicates for each tissue; minimum 1 bp overlap between biological replicates and between peak sets for each tissue. (**c**) Normalized read depth of ATAC-seq libraries at two genes: Interleukin 16 (IL16), a modulator of T cell activation, and transgelin (TAGLN), an actin cross-linking protein.

To confirm the broader applicability of this modified ATAC-seq protocol, the technique was also applied to lung, muscle, and spleen collected from adult male Yorkshire pigs (Michigan State University) as part of the FAANG pilot project at UC Davis. For all libraries, over 100,000 NFR were called, which consistently overlapped with the active histone modifications H3K27ac, H3K4me1, and H3K4me3 (Fig. 6a; ChIP-seq data generated as part of the UC Davis FAANG pilot project). Over 20% of reads fell within peaks, with reads distributed across both distal and promoter NFR (Fig. 6b), which marked about a third of promoters, on average (Fig. 6c). Tissue replicates clustered together based on read alignments (Fig. 7a), and NFR were consistent between biological replicates, with 68%, 87%, and 64% of peaks detected in both replicates of lung, muscle, and spleen, respectively.

**Figure 8:**
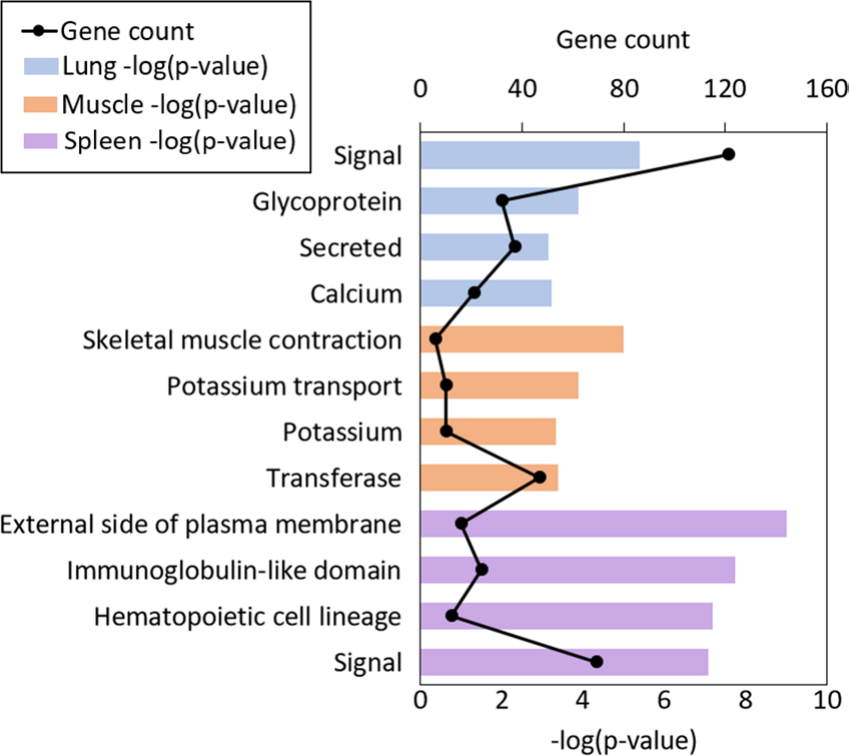
Top four enriched functional terms for genes whose promoters were marked by tissue-specific open chromatin in pig lung, muscle, and spleen. All functional terms were enriched with a FDR < 0.05, and include Gene Ontology terms, KEGG pathways, and UP_KEYWORDS.

Comparing sets of NFR revealed both common and tissue-specific NFR (Fig. 7b), such as those observed at genes with tissue-specific functions (Fig. 7c). Muscle-specific NFR were enriched for binding motifs of various myocyte enhancer factors, whereas NFR that were common to all three tissues were primarily enriched for the CTCF motif and promoter-specific sequences (Table 2). Genes with tissue-specific NFR in their promoters were similarly enriched for tissue-specific functions, with muscle-specific NFR marking genes related to muscle contraction and ion transport, and spleen-specific NFR marking genes related to immune function (Fig. 8). These data suggest that this modified ATAC-seq technique may be broadly applied across species and tissues to profile chromatin accessibility.

**Table 2:** Top 5 enriched known motifs found in tissue-specific and ubiquitous NFR, identified in pig lung, muscle, and spleen.

Common NFR		Lung-specific NFR		Muscle-specific NFR		Spleen-specific NFR	
Motif	P-value	Motif	P-value	Motif	P-value	Motif	P-value
CTCF	1E-2149	Foxa2	1E-209	Mef2c	1E-1065	PU.1	1E-675
BORIS	1E-1327	FOXA1	1E-185	Mef2d	1E-969	ETS1	1E-640
Sp1	1E-535	EKLF	1E-175	Mef2a	1E-783	Etv2	1E-588
ETS	1E-363	CEBP	1E-164	Six1	1E-679	CTCF	1E-545
ELF1	1E-285	Klf4	1E-155	Fra1	1E-495	Fli1	1E-518

## Discussion

While ATAC-seq has been used extensively to profile open chromatin in cell lines, its applicability to other sample types, such as frozen tissues, has been limited. Another modified ATAC-seq protocol – Omni-ATAC – reportedly detected 76% of DHS in a cell line^10^. However, Omni-ATAC data generated from frozen tissue was not compared to reference DNase-seq data, so conclusions could not be drawn about the consistency between Omni-ATAC and DNase-seq from frozen tissue samples. Additionally, concerns about possible degradation of the nuclear architecture during flash-freezing suggest that slow-freezing, or cryopreservation, may be the ideal long-term storage method for samples intended for ATAC-seq^11^. Through systematic modifications to ATAC-seq, chromatin accessibility data for chicken lung were generated from cryopreserved nuclei preparations and compared with DNase-seq, ChIP-seq, and RNA-seq data also available for chicken lung. These ATAC-seq data detected 55% of DHS in chicken lung at a depth of 40 million reads, consistent with the original ATAC-seq protocol, which detected 46% of DHS in a human cell line^8^.

Cell quality and thorough lysis appeared to be the most important criteria for generating high-quality ATAC-seq libraries from cryopreserved nuclei preparations. This was exemplified by the observation that subjecting the sample to an additional cell lysis step improved the specificity of ATAC-seq peaks to actively expressed genes. In addition, doubling the tagmentation time and sequencing only the subnucleosomal fraction of libraries substantially improved the signal-to-noise ratio after mapping, ultimately resulting in ATAC-seq data that meets ENCODE standards. Attempts to evaluate library quality before sequencing were largely uninformative. Sequencing data revealed that the presence of a nucleosomal laddering pattern did not necessarily indicate a good-quality ATAC-seq library. In fact, strong laddering patterns were evident in libraries that demonstrated very low signal-to-noise ratios (as low as 3% of reads in peaks). Ultimately, we found that the best way to gauge library quality was to generate preliminary sequencing data at a low depth of about 5 million mapped reads, and assess the signal-to-noise ratio by the fraction of reads falling in peaks (Figs. 3c and 6c), which should exceed 10% for good quality libraries.

Overall, we conclude that (1) sample preparation (cell quality and thorough lysis) is critical for generating high quality libraries, (2) nucleosomal laddering is not a consistent indicator of library quality, and (3) sequencing only the subnucleosomal fraction of libraries considerably improved the signal-to-noise ratio after mapping. Following ATAC-seq optimization, regions enriched for ATAC-seq reads captured more DHS and regions with active histone modifications. Consolidating these findings yields a modified ATAC-seq protocol capable of generating high-quality chromatin accessibility data from cryopreserved nuclei preparations. These results broaden the applicability of ATAC-seq to cryopreserved nuclei preparations from tissues, which will benefit current efforts to annotate regulatory elements in a wider range of samples and species.

## Materials and Methods

### Tissue collection, processing, and cryopreservation of nuclei preparations

Lung tissue was harvested from inbred adult roosters (UCD003 line). One gram of tissue was minced with a razor blade, transferred to a gentleMACS C tube with 10 mL of sucrose buffer (250 mM D-Sucrose, 10 mM Tris-HCl (pH 7.5), 1 mM MgCl_2_; 1 protease inhibitor tablet per 50 mL solution just prior to use), and homogenized using the gentleMACS Dissociator Program ‘E.01c Tube’, twice. Homogenate was filtered using a 100 uM Steriflip Vacuum Filter system. Volume was brought up to 9.9 mL with sucrose buffer, and 1.1 mL DMSO was added to achieve a 10% final concentration. Solution was aliquoted into cryotube vials, frozen overnight at −80 °C in a Nalgene Cryo 1 °C/min Freezing Container, and then stored at −80 °C long-term.

All animal experimental protocols were approved by the University of California Davis Animal Care and Use Committee (IACUC). All methods were performed in accordance with the relevant guidelines and regulations.

### Final modified ATAC-seq protocol (Iteration 3; for libraries 3-d-200K, 3-e-200K, and all pig libraries)

Reagents and Materials

- Fresh tissue
- Dimethyl Sulfoxide (DMSO; Sigmal Aldrich, cat. no. D2650)
- Sucrose buffer
  *250 mM D-Sucrose (Fisher Scientific, cat. no. BP220-1)*
  *10 mM Tris-HCl, pH 7.5 (Mediatech Inc., cat. no. 46-030-CM)*
  *1 mM MgCl2 (Ambion, cat. no. AM9530G)*
  *Molecular Biology Grade sterile H2O to 500 ml*
- Phosphate buffered saline (PBS; ThermoFisher, cat. no. 10010023)
- Cell lysis buffer
  *10 mM Tris-HCl, pH 7.4*
  *10 mM NaCl (Sigma Aldrich, cat. no. S7653)*
  *3 mM MgCl2*
  *0.1% (v/v) Igepal CA-630 (Sigma Aldrich, cat. no. I8896)*
  *Store up to 1 week at 4 °C*
- TD (2x reaction buffer from Nextera kit; Illumina, cat. no. FC-121-1030)
- TDE1 (Nextera Tn5 Transposase from Nextera kit; Illumina, cat. mo. FC-121-1030)
- Nuclease-free H_2_O (available from various molecular biology suppliers)
- MinElute PCR Purification Kit (Qiagen, cat. no. 28004)
- 25 µM PCR Primer 1 [custom-synthesized by Integrated DNA Technologies (IDT); sequences provided in Buenrostro *et al*. (2013)]
- 25 µM PCR Primer 2 [custom-synthesized by Integrated DNA Technologies (IDT); sequences provided in Buenrostro *et al*. (2013)]
- SsoFast™ EvaGreen® Supermix (Bio-Rad Laboratories, cat. no. 1725201)
- Refrigerated swinging bucket centrifuge
- 1.5-ml Eppendorf tubes
- 0.2-ml PCR tubes
- PCR thermal cycler
- Bioanalyzer High-Sensitivity DNA Analysis kit (Agilent)
- PippinHT system, 3% agarose DNA size selection cassette (100–250 bp)

Protocol

1. Prior to ATAC-seq:
  - Pre-chill centrifuge with swinging bucket rotor to 4 °C
  - Prepare ATAC-seq cell lysis buffer
  - Thaw nuclei preparations on ice
2. Centrifuge preparations for 5 min, 500 rcf, 4 °C
3. Aspirate supernatant and resuspend pellet in 1 mL cold PBS
4. Centrifuge for 5 min, 500 rcf, 4 °C
5. Aspirate supernatant and resuspend pellet in 1 mL cold ATAC-seq cell lysis buffer
6. Centrifuge 10 min, 500 rcf, 4 °C
7. Aspirate supernatant and resuspend pellet cold PBS for counting on hemocytometer
8. Aliquot cells to 1.5 mL Eppendorf tubes
9. Centrifuge 5 min, 500 rcf, 4 °C
10. Carefully aspirate supernatant and resuspend pellet in 50 µL transposition mix (25 uµL TD buffer, 2.5 µL TDE1 enzyme, 22.5 µL ddH2O)
11. Incubate nuclear pellet with transposition mix for 60 min, 37 °C, 300 rpm.
12. Purify transposed DNA with MinElute PCR purification kit (elute DNA with 10 µL Buffer EB)
13. Add 40 µL PCR master mix (25.4 µL SsoFast™ EvaGreen® Supermix, 13 µL ddH_2_O, 0.8 µL 25 µM Primer 1, 0.8 25 µM Primer 2) to 10 µL eluted DNA and cycle as follows:
  1 × 5 min 72 C
  30 sec 98 C
  5 × 10 sec 98 C
  30 sec 63 C
  1 min 72 C
14. Subject PCR reaction to 5 additional cycles (for a total of 10 cycles). Optionally, an aliquot of the PCR reaction may be subjected to qPCR to determine the optimal number of additional cycles to minimize PCR bias in final library, with the additional number of cycles calculated as those needed to reach 1/3 of the maximum R_n_ value determined by qPCR (see Buenrostro *et al*. (2013) for additional details on using qPCR to determine optimal PCR cycles).
  1 × 30 sec 98 C
  5 × 10 sec 98 C
  30 sec 63 C
  1 min 72 C
15. Purify libraries using MinElute PCR purification kit (elute with 10 µL Buffer EB)
16. Quantify libraries and run traces on Agilent Bioanalyzer High Sensitivity DNA chip
17. Size-select libraries for subnucleosomal fragments (150–250 bp) on the PippinHT system using a 3% cassette
18. Submit libraries for sequencing to a depth of ~50 million reads per sample (NextSeq, paired-end 40 bp reads)

### Modified ATAC-seq protocol (Iteration 1; for library 1-a-50K)

Cryopreserved nuclei preparations were thawed on ice, pelleted (600 × g for 10 min at 4 °C), resuspended in sucrose buffer (250 mM D-Sucrose, 10 mM Tris-HCl (pH 7.5), 1 mM MgCl_2_), filtered through a 20 µm Steriflip Vacuum filter system, pelleted again (500 × g for 5 min at 4 °C), washed with PBS (500 × g for 5 min at 4 °C), and resuspended in PBS for counting. Nuclei concentration was determined by counting on a hemocytometer, an aliquot of 50,000 nuclei was pelleted in a 1.5 mL Eppendorf tube (500 × g for 5 min at 4 °C), and the nuclear pellet was incubated in 50 µL transposition mix (25 µL TD buffer, 2.5 µL TDE1 enzyme, 22.5 µL ddH_2_O) for 30 minutes at 37 °C (300 rpm). Transposed DNA was then purified with the MinElute PCR Purification kit (DNA was eluted with 10 µL Buffer EB). DNA was then amplified by PCR. 40 µL PCR master mix (25.4 µL SsoFast™ EvaGreen® Supermix, 13 µL ddH_2_O, 0.8 µL 25 µM Custom Primer 1, 0.8 µL 25 µM Custom Primer 2) was added to 10 µL eluted DNA and cycled as follows: 1 ×[5 min at 72 °C, 30 sec at 98 °C], then 5 ×[10 sec at 98 °C, 30 sec at 63 °C, 1 min at 72 °C]. To determine the optimal number of PCR cycles, 5 µl of the PCR reaction was added to 10 µl of qPCR master mix (6.45 µl SsoFast™ EvaGreen® Supermix, 3.25 µl ddH_2_O, 0.15 µl 25 µM Custom Primer 1, 0.15 µl 25 µM Custom Primer 2) and cycled on a qPCR instrument as follows: 1 ×[30 sec at 98 °C], then 20 ×[10 sec at 98 °C, 30 sec at 63 °C, 1 min at 72 °C]. Linear fluorescent intensity (R_n_) was plotted against cycle to determine the additional number of cycles needed (*N*): the number of cycles required to reach one third of the maximum R_n_. In this case it was determined that 7 additional cycles were needed. The remaining 45 µl of the PCR reaction was then cycled as follows: 1 ×[30 sec at 98 °C], then (*N* = 7) × [10 sec at 98 °C, 30 sec at 63 °C, 1 min at 72 °C]. Libraries were then purified with the MinElute PCR Purification kit and eluted in 10 µL Buffer EB. Libraries were assessed for fragment length distribution on a Bioanalyzer High Sensitivity DNA Chip (Agilent Genomics) and submitted for paired-end 100 bp sequencing on the HiSeq3000 platform.

### Modified ATAC-seq protocol (Iteration 2; for libraries 2-b-50K, 2-b-500K, 2-c-100K, 2-c-200K, 2-c-500K-1, and 2-c-500K-2)

Cryopreserved nuclei preparations were thawed on ice, and carefully layered on top of a sucrose gradient with 0.3 M sucrose buffer on top and 1.8 M sucrose buffer on the bottom (0.3 M or 1.8 M D-Sucrose, 10 mM Tris-HCl (pH 7.5), 1 mM MgCl_2_) and centrifuged (4500 × g for 45 min at 4 °C). Pelleted nuclei were resuspended in PBS for counting. Nuclei concentration was determined by counting on a hemocytometer, an aliquot of 50,000–500,000 nuclei was pelleted in a 1.5 mL Eppendorf tube (500 × g for 5 min at 4 °C), and the nuclear pellet was incubated in 50 µL transposition mix (25 µL TD buffer, 2.5 µL TDE1 enzyme, 22.5 µL ddH_2_O) for 30 minutes at 37 °C (300 rpm). Transposed DNA was then purified with the MinElute PCR Purification kit (DNA was eluted with 10 µL Buffer EB). DNA was then amplified by PCR. 40 µL PCR master mix (25.4 µL SsoFast™ EvaGreen® Supermix, 13 µL ddH_2_O, 0.8 µL 25 µM Custom Primer 1, 0.8 µL 25 µM Custom Primer 2) was added to 10 µL eluted DNA and cycled as follows: 1 ×[5 min at 72 °C, 30 sec at 98 °C], then 10 ×[10 sec at 98 °C, 30 sec at 63 °C, 1 min at 72 °C]. Libraries were then purified with the MinElute PCR Purification kit and eluted in 10 µL Buffer EB. If a library was not concentrated enough for sequencing after 10 cycles of amplification, library complexity was a concern, and library preparation was repeated. Libraries were assessed for fragment length distribution on a Bioanalyzer High Sensitivity DNA Chip (Agilent Genomics), and submitted for paired-end 40 bp sequencing on the NextSeq platform.

### ATAC-seq sequencing data analysis

Raw sequencing data were trimmed using Trim Galore! (0.4.0), a wrapper around Cutadapt (1.12)^14^ (-a CTGTCTCTTATA –length 10). Trimmed reads were aligned to the Galgal5 assembly^15^ or Sscrofa10.2^16^ assembly using BWA (0.7.17)^17^ aln (-q 15) and sampe (-a 2000). Aligned reads were filtered to remove PCR duplicates using Picard Tools (2.7.1). Mitochondrial and low quality (q < 15) alignments were then removed using SAMtools^18^ (1.9). Finally, MACS2 (2.1.1)^19^ was used to call broad peaks (-q 0.05 -B –broad –nomodel –shift −100 –extsize 200).

### DNase-seq sequencing data analysis

Raw sequencing data were trimmed using Trim Galore! (0.4.0), a wrapper around Cutadapt (1.8.3)^14^ (-a AGATCGGAAGAGC). Trimmed reads were aligned to the Galgal5 assembly^15^ using BWA (0.7.15)^17^ aln (-q 15) and sampe (-a 2000). Aligned reads were filtered to remove PCR duplicates using Picard Tools (2.7.1). Low quality (q < 15) alignments were then removed using SAMtools (1.9)^18^. Finally, MACS2 (2.1.0)^19^ was used to call broad peaks (-q 0.05 -B –broad).

### ChIP-seq sequencing data analysis

Raw sequencing data were trimmed using Trim Galore! (0.4.0), a wrapper around Cutadapt (1.8.3)^14^ (-a AGATCGGAAGAGC). Trimmed reads were aligned to the Galgal5 assembly^15^ or Sscrofa10.2^16^ assembly using BWA (0.7.15)^17^ aln (-q 15) and sampe (-a 2000). PCR duplicates were marked using Picard Tools (2.7.1). Low quality (q < 15) alignments were then removed using SAMtools (1.9)^18^. Finally, MACS2 (2.1.0)^19^ was used to call broad peaks for H3K4me1 (-q 0.1 -B–broad–SPMR–keep-dup all–nomodel–extsize 8) and narrow peaks for H3K4me3 (-q 0.01 -B–SPMR–keep-dup all –nomodel –extsize 204) and H3K27ac (-q 0.01 -B–SPMR–keep-dup all–nomodel–extsize 220).

### RNA-seq sequencing data analysis

Raw reads were trimmed using Trim Galore! (0.4.4), a wrapper around Cutadapt (1.16)^14^ (-a AGATCGGAAGAGC). Trimmed reads were aligned to the Galgal5 assembly^15^ using STAR^20^ (2.5.3) (–outFilterType BySJout–outFilterMultimapNmax 20–alignSJoverhangMin 8–alignSJDBoverhangMin 1–outFilterMismatchNmax 999–alignIntronMin 20). Raw read counts were calculated using summarizeOverlaps from the GenomicRanges Bioconductor package^21^ (mode = “Union”, fragments = T). Raw counts were normalized using the variance-stabilizing transformation method from the DESeq2^22^ R package.

### Read alignment length distributions

Observed template length was extracted from alignment (BAM) files using SAMtools^18^ (1.9).

### Calculation of the Fraction of Reads in Peaks (FRiP) score

The BEDTools^23^ intersect function was used to identify all reads in peaks: reads that overlapped an ATAC-seq peak by at least 20% of the read length. Reads in peaks were further classified as overlapping a promoter (2 kb upstream of TSS; minimum overlap 50% of read length) or as being distal. The FRiP score was calculated by dividing reads in peaks by total reads used for calling peaks.

### ATAC-seq peak overlap with other ATAC-seq peaks, DHS and ChIP-seq peaks

Overlap was determined using the BEDTools^23^ package’s intersect function with default arguments (minimum 1 bp overlap).

### Gene expression versus promoter chromatin state

Genes were classified as open or closed based on whether or not an ATAC-seq peak overlapped their promoter (2 kb upstream of the TSS). Overlap was determined using the BEDTools^23^ package’s intersect function with default arguments (minimum 1 bp overlap).

### Normalized read coverage plots

Normalized read coverage plots were generated using the *profile* function from the Fluff^24^ package (3.0.2).

### Genomic localization of ATAC-seq peaks

Peaks were sorted by genomic location using the BEDTools^23^ intersect function, based on minimum 50% overlap with promoters (2 kb upstream of TSS), exons, or introns. Lack of overlap with any of these features classified a peak as distal.

### Binding motif enrichment analysis

Sets of peaks in interval format (BED files) were evaluated for binding motif enrichment using the findMotifsGenome.pl script from HOMER^25^ (v4.8) and the top known motifs, based on p-value, were reported.

### Principal components analysis (PCA) of pig tissue ATAC-seq alignments

Alignment files were converted to bigwig format and normalized by reads per kilobase million (RPKM) with the bamCoverage function from the deepTools^26^ suite (2.5.0). Bigwig files were used to generate a PCA plot using the plotPCA function from the deepTools suite (2.5.0).

### Functional annotation enrichment analysis

Gene sets were submitted to DAVID^27,28^ (v6.8) to identify enriched biological functions using default settings. Resulting functional annotation terms were sorted by FDR, and the most significant terms reported.

## Supplementary information

Supplementary Figures

ATACSeq_Pig_Lung_Combined_Peaks

ATACSeq_Pig_Muscle_Combined_Peaks

ATACSeq_Pig_Spleen_Combined_Peaks

ATACSeq_Chicken_Lung_Combined_Peaks

DNaseSeq_Chicken_Lung_Peaks.

H3K4me1_Chicken_Lung_Peaks

H3K4me3_Chicken_Lung_Peak.

H3K27ac_Chicken_Lung_Peaks.

Genes with promoters marked by an ATAC-seq peak in pig tissues

## Data Availability

The raw ATAC-seq data generated from chicken lung for this study are available from the ATAC-seq data generated for this study are available from the Sequence Read Archive (SRA) under accession PRJNA605842 (https://www.ncbi.nlm.nih.gov/bioproject/PRJNA605842). All other raw sequencing data, including DNase-seq, RNA-seq, ChIP-seq, and ATAC-seq from pig tissues, are available from the European Nucleotide Archive under project ID PRJEB14330 (https://www.ebi.ac.uk/ena/data/view/PRJEB14330).
