# Supplementary material for: Systematic alteration of ATAC-seq for profiling open chromatin in cryopreserved nuclei preparations from livestock tissues
Source: Sci Rep. 2020 Mar 23;10:5230. doi: 10.1038/s41598-020-61678-9 (PMC7089989; doi:10.1038/s41598-020-61678-9)
Supplement: Supplementary file 1 — Supplementary Figures [file 41598_2020_61678_MOESM1_ESM.docx]

**Systematic alteration of ATAC-seq for profiling open chromatin in cryopreserved nuclei preparations from livestock tissues**

Halstead MM, Kern C, Saelao P, Chanthavixay G, Wang Y, Delany ME, Zhou H, Ross PJ

Department of Animal Science, University of California, Davis, Davis, CA

**Supplementary information**


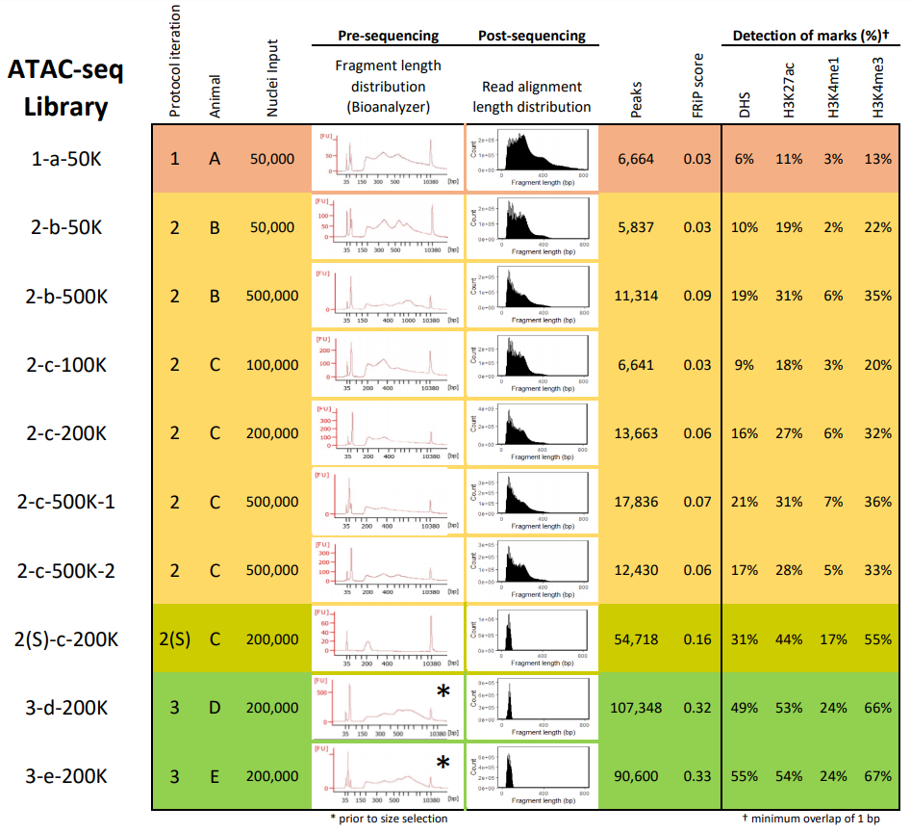


**Supplementary Figure 1. Summary of libraries for each ATAC-seq protocol iteration, produced from cryopreserved chicken lung nuclei.** Row colors indicate different iterations of the ATAC-seq protocol. Library names indicate “Protocol iteration–Animal–Nuclei input(–Replicate)”. Progressive changes to the protocol improved signal (Fraction of Reads in Peaks (FRiP) score) and improved overlap with DNase-I Hypersensitive Sites (DHS) and active histone modifications (minimum 1 bp overlap).


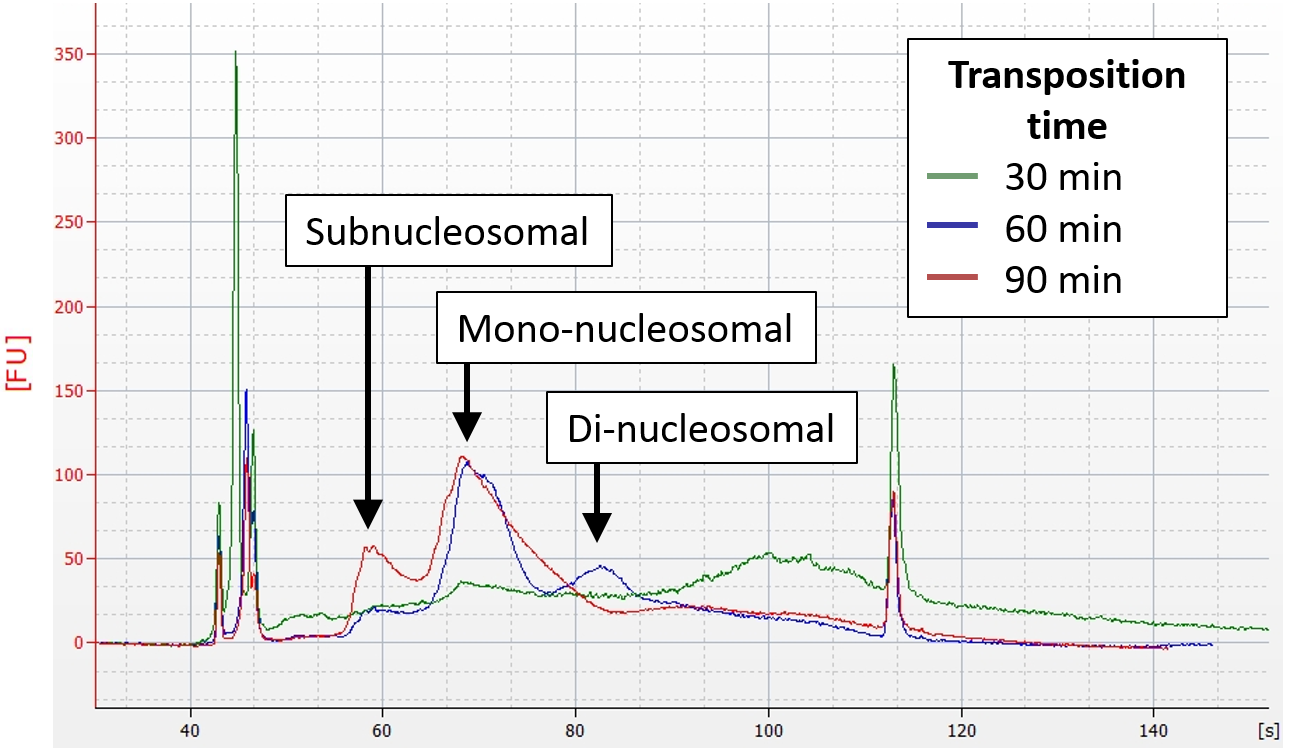


**Supplementary Figure 2. Varying transposition time affects library fragment length distribution.** Bioanalyzer trace shows that the fragment length distribution of ATAC-seq libraries is shifted towards subnucleosomal length fragments when transposition time is increased from 30 to 90 minutes.


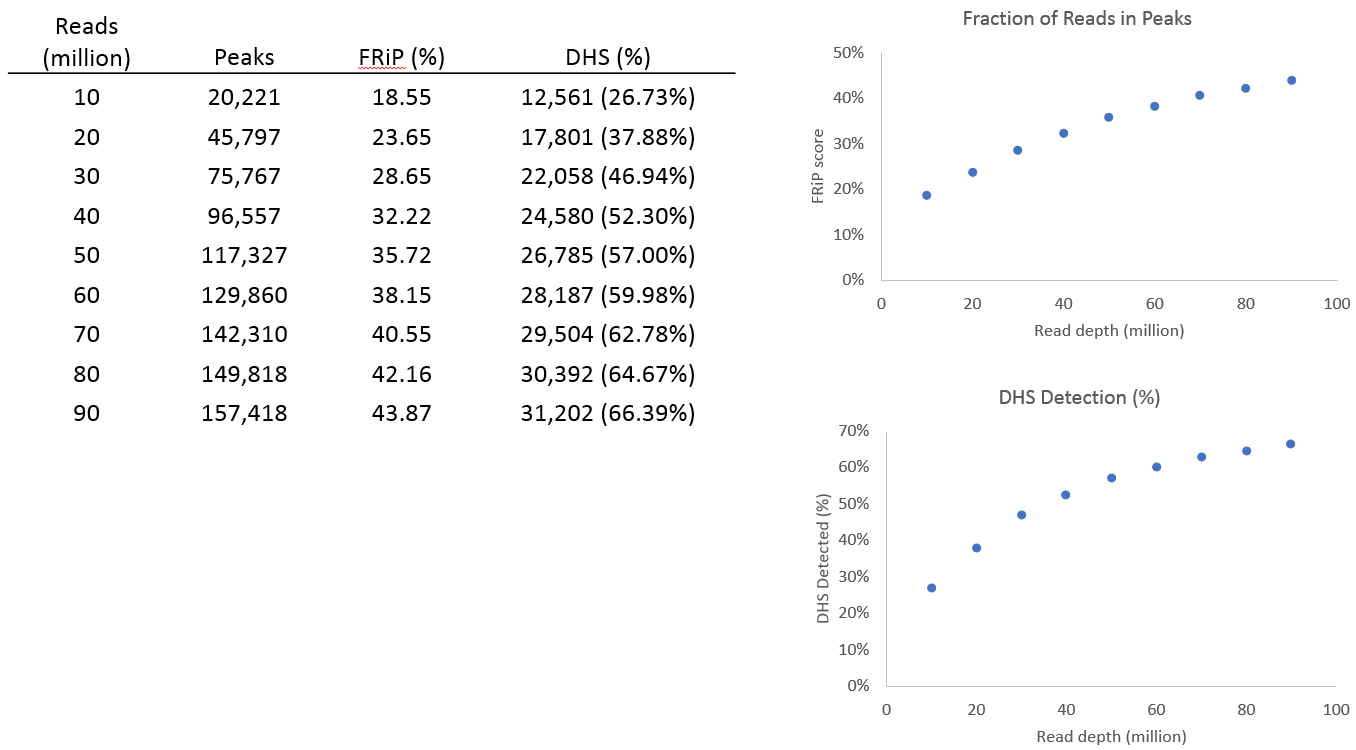
**Supplementary Figure 3. The combined set of *3-d-200k* and *3-e-200k* (uniquely-mapped non-mitochondrial monoclonal) was subsampled and used for peak calling to determine optimal sequencing depth for future experiments.** Number of peaks called, fraction of reads in peaks (FRiP), and detection of DHS (min 1 bp overlap) started to plateau after 50 million reads.

**Supplementary Datasets**

ATAC-seq peak interval files:

1. ATACSeq_Pig_Lung_Combined_Peaks.bed
2. ATACSeq_Pig_Muscle_Combined_Peaks.bed
3. ATACSeq_Pig_Spleen_Combined_Peaks.bed
4. ATACSeq_Chicken_Lung_Combined_Peaks.bed

DNase-seq and ChIP-seq peak interval files:

1. DNaseSeq_Chicken_Lung_Peaks.bed
2. H3K4me1_Chicken_Lung_Peaks.bed
3. H3K4me3_Chicken_Lung_Peaks.bed
4. H3K27ac_Chicken_Lung_Peaks.bed

List of genes with promoters marked by an ATAC-seq peak in pig tissues
